# The Relationship of the Pathologist to the Surgeon

**Published:** 1905-10-14

**Authors:** 


### The Relationship of the Pathologist to the Surgeon.

The relationship of pathology to surgery fortunately tends to become closer year by year, and the closer it becomes the better it is for surgery. Formerly there was no pathology, and before that there was not even morbid anatomy; yet surgery as an art made great strides in advance without any knowledge of pathological processes. Cheselden, with only an elementary knowledge of anatomy, brought to perfection the operation of cutting for stone; Liston, and the surgeons who practised just before the introduction of anæsthetics were perhaps the most dexterous operators the world has ever seen, for they combined boldness with speed. We owe to John Hunter and the school which he taught the knowledge that morbid anatomy and comparative anatomy might be useful in the practice of surgery. The Hunterian school of surgery lasted until the end of the nineteenth century, and produced a series of scientific surgeons, whose work was of the greatest value. Their work was based at first upon anatomy, but in later years the utility of the microscope as an aid both to diagnosis and treatment was fully recognised, and the most important advances were made in connection with the treatment of the various forms of tumour. The rise of bacteriology opened up a new field of work for surgeons who were inclined to the scientific side of their profession. It taught them much about the causes and nature of inflammation, and it showed them how the process of repair can be best promoted. Incidentally, too, it has shown that there may be a vast and unexplored field of prophylactic surgery whereby tissues can be rendered immune to various infective agents, the results of whose activity have now to be treated by the knife. The labours of Pasteur were amplified and applied by Lister, but the methods of these masters have been still further modified and improved by surgeons in every part of the civilised world. The first result obtained was an increased safety in operating, and this, in turn, has led to an enormous increase in the number of operations performed. The patients themselves have been quick to seize these advantages, and where it used to be difficult to gain consent for an operation, it is now not only easy, but the sufferer actually comes to the surgeon asking for relief. Such an attitude on the part of the public is fraught with good, for the cases are seen earlier, and in the larger towns, at any rate, enormous tumours, strangulated herniæ, which have become gangrenous and joints diseased beyond recovery, are now almost things of the past.

Surgeons until recently have always been their own pathologists, but with the growth of bacteriology and the increase in the number of operations it is becoming impossible for one man to act as surgeon and at the same time to do pathology. Surgery and pathology, therefore, are becoming divorced, as is well seen if two lists of the officers of the Pathological Society of London be compared. Ten years ago the president, one secretary, and at least half the council were surgeons attached to the great hospitals of London; now, out of a council of thirty, not a single one is practising as a surgeon. This divorce is not altogether good, either for surgeons or for pathologists. The surgeon has to accept the scientific basis of his profession at second hand, whilst the pathologist, seeing little or nothing of the clinical side of surgery, is unable to be as practical as his predecessor who spent much of his time in the wards. In nothing is this fault more clearly observed than in the histological reports received from the various pathological laboratories upon tumours sent for examination. The pathological histologist, acting in all good faith, bases his statements entirely on the microscopical appearances, but a surgeon trained in pathology discounts the value of the report thus obtained, for he knows that the clinical course in many cases does not agree with the histological results. An attempt is now being made to avoid this divergence between the clinical and pathological aspects of a case, for there is growing up a class of consulting pathologists who, for an adequate fee, will accompany a surgeon to a difficult case, and give him the benefit of his trained opinion. It appears as if the opening for such a class of practitioners will be of increasing importance. A section of a doubtful tumour can now be made whilst the surgeon is actually operating, and the extent of the operation can be determined by the knowledge thus obtained. An obscure abdominal case can often be cleared up by a careful chemical examination of the gastric or intestinal contents, whilst an examination of the blood may give the most important results. The information obtained is of greater value than if the material had simply been sent to a laboratory, for the pathologist has seen and examined the patient with the surgeon, and has been able to draw his conclusions in the light of his own experience. Many qualities are required of such a pathologist—some innate, others acquired—but as a surgeon has less and less pathological training, the consulting pathologist should have a larger and larger field of work.

